# Supplementary material for: Outcome After 68Ga-PSMA-11 versus Choline PET-Based Salvage Radiotherapy in Patients with Biochemical Recurrence of Prostate Cancer: A Matched-Pair Analysis
Source: Cancers (Basel). 2020 Nov 16;12(11):3395. doi: 10.3390/cancers12113395 (PMC7698293; doi:10.3390/cancers12113395)
Supplement: Supplementary file 1 [file cancers-12-03395-s001.pdf]

Supplementary Material: Outcome After 68Ga-PSMA-11 versus Choline PET-Based Salvage Radiotherapy in Patients with Biochemical Recurrence of Prostate Cancer: a Matched-Pair Analysis

Nina-Sophie Schmidt Hegemann, Paul Rogowski, Chukwuka Eze, Christian Schäfer, Christian Stief, Sebastian Lang, Simon Spohn, Rieke Steffens, Minglun Li,. Christian Gratzke, Wolfgang Schultze-Seemann, Harun Ilhan, Wolfgang Peter Fendler, Peter Bartenstein, Ute Ganswindt, Alexander Buchner, Anca-Ligia Grosu, Claus Belka, Philipp T. Meyer, Simon Kirste and Constantinos Zamboglou

Table S1. Patient characteristics after propensity score matching.

	Choline Cohort	PSMA Cohort	*p* Value
Number (%)	136 (100)	136 (100)	
Age in y (median, range)	69 (47–82)	72 (49–83)	n.s.
Initial PSA in ng/mL (median, range)	9.6 (1.1–281)	10 (2.4–238)	n.s.
pT stage (%)			**
2	51 (37.5)	65 (47.8)	
3a	38 (27.9)	38 (27.9)	
3b	29 (21.3)	30 (22.1)	
4	5 (3.7)	2 (1.5)	
unknown	13 (9.6)	1 (0.7)	
Gleason score (%)			n.s.
6	14 (10.3)	5 (3.6)	
7a	36 (26.5)	42 (30.9)	
7b	44 (32.4)	38 (27.9)	
8	21 (15.4)	24 (17.6)	
≥9	16 (11.7)	25 (18.4)	
unknown	5 (3.7)	2 (1.5)	
NCCN risk group (%)			n.s.
low risk	3 (2.2)	6 (4.4)	
intermediate risk	79 (58.1)	74 (54.4)	
high risk	54 (39.7)	56 (41.2)	
unknown	0	0	
R status (%)			**
R0	68 (50)	82 (60.3)	
R1 or R2	44 (32.4)	49 (36)	
Rx	24 (17.6)	5 (3.7)	
pN status (%)			n.s.
pN0	118 (86.8)	118 (86.8)	
pN1	18 (13.2)	18 (13.2)	
pNx	0	0	
PSA persistence after surgery (%)			n.s.
yes	40 (29.4)	42 (30.9)	
no	96 (70.6)	94 (69.1)	
PSA before imaging in ng/mL (range, median)	0.6 (0.7–30)	0.58 (0.14–31.3)	n.s.
PET findings (%)			n.s.
Positive PET findings	78 (57.4)	80 (58.8)	
No PET findings	58 (42.6)	56 (41.2)	
Locally recurrent disease	64 (47.1)	50 (36.8)	
Pelvic lymph nodes	27 (19.9)	45 (33.1)	

Metastatic disease	8 (5.9)	10 (7.4)	
PSA before sRT in ng/mL (range, median)	0.83 (0.2–17.6)	0.65 (0.1–27.5)	n.s.

Table S2. Treatment characteristics after propensity score matching.

	**Choline cohort**	**PSMA Cohort**	***p* Value**
Number (%)	136 (100)	136 (100)	
ADT			**
yes	27 (19.9)	52 (38.2)	
no	109 (80.1)	84 (61.2)	
Median duration ADT in months			n.s
≤6 months	14 (10.3)	38 (27.9)	
>6 months	9 (6.6)	12 (8.8)	
Unknown duration (n, %)	4 (3)	2 (1.5)	
RT dose prostatic fossa in Gy (median, range)	70.2 (56–72)	66.6 (59.4–74)	*
RT to elective pelvic lymphatics			*
yes	33 (24.3)	51 (37.5)	
no	103 (75.7)	85 (62.5)	
RT dose to elective pelvic lymphatics in Gy (median, range)	50.4 (36–54)	50.4 (45–50.4)	n.s.
RT boost to pelvic lymph nodes			**
yes	26 (19.1)	45 (33.1)	
no	110 (80.9)	87 (66.9)	
RT dose to pelvic lymph nodes in Gy (median, range)	55 (50.4–66)	57.6 (45–57.6)	n.s.
RT to distant metastases			n.s.
yes	8 (5.9)	10 (7.4)	
no	128 (94.1)	126 (93.6)	
RT dose to distant metastases in Gy (median, range)	35 (30–54)	40 (34–64)	n.s.
Follow-up time in months (median, range)	27 (1–108)	22 (2–51)	***
Biochemical recurrent disease			n.s.
yes	51 (37.5)	48 (35.3)	
no	85 (62.5)	88 (64.7)	

Table S3. Cox regression analysis of the entire cohort after the exclusion of patients with ongoing ADT at least 3 months prior to last follow-up examination.

	**Cox Ratio (95% CI)**	***p* Value**		
**Indication RT (persistence vs. salvage)**	**1.7 (1.2–2.3)**	**0.003**		
Risk group NCCN (LR vs. IR vs. HR)	1.3 (0.9–1.8)	0.066		
**R status (R0 vs. R1)**	**0.7 (0.5–0.99)**	**0.049**		
**Gleason score (6 vs. 7 vs. 8)**	**1.4 (1.02–1.8)**	**0.035**		
**pT (2 vs. 3 + 4)**	**1.5 (1.1–2.2)**	**0.19**		
**pN (0 vs. 1)**	**1.6 (1.2–2.2)**	**0.002**		
Time between surgery and PET imaging (days, continuous variable)	1.004 (1.001–1.007)	0.253		
**Tracer (PSMA vs. cholin)**	**1.5 (1.1–2.2)**	**0.02**		
**PSA before PET (< / >0.5 ng/mL))**	**1.7 (1.2–2.4)**	**0.003**		
**PSA before PET (ng/mL, continuous variable)**	**1.1 (1.06–1.12)**	**<0.001**		
**PSA before sRT (< / >0.5 ng/mL)**				
**PSA before sRT (ng/mL, continuous variable)**	**1.1 (1.05–1.12)**	**<0.001**		
**Initial PSA (ng/mL, continuous variable)**	**1.004 (1.001–1.007)**	**0.025**		
**PET-positive findings (yes vs. no)**	**1.7 (1.2–2.5)**	**0.003**		
Local recurrence in PET (yes vs. no)	1.1 (0.8–1.6)	0.466		
**Pelvic lymph nodes in PET (yes vs. no)**	**1.9 (1.3–2.6)**	**<0.001**		
**Distant metastases in PET (yes vs. no)**	**2.9 (1.8–4.6)**	**<0.001**		
RT dose prostatic fossa (< / > 66 Gy)	1.1 (0.6–2.1)	0.725		
ADT (yes vs. no)	0.9 (0.7–1.4)	0.912		
**Multivariate Cox regression analysis**				
	Cox ratio	95% CI		*p*
		lower	upper	value
Indication RT (persistence vs. salvage)	1.168	0.777	1.758	0.455

Risk group NCCN (LR vs. IR vs. HR)	0.817	0.489	1.365	0.441
Gleason score (6 vs. 7 vs. 8)	1.183	0.720	1.942	0.507
pT (2 vs. 3 + 4)	1.247	0.799	1.948	0.331
pN (0 vs. 1)	1.226	0.838	1.793	0.294
Tracer (PSMA vs. Cholin)	1.115	0.738	1.685	0.605
**PSA before PET (ng/mL, continuous variable)**	**1.067**	**1.022**	**1.115**	**0.003**
Initial PSA (ng/mL, continuous variable)	1.000	0.995	1.004	0.902
PET-positive findings (yes vs. no)	1.169	0.720	1.897	0.529

Bold: statistically significant values. CI: confidence interval.
